# Preoperative imaging of deep endometriosis: pitfalls of a diagnostic test before surgery

**Published:** 2021-01-08

**Authors:** PR Koninckx, A Deslandes, A Ussia, A Di Giovanni, G Hanan, M Tahlak, L Adamian, J Keckstein, A Wattiez

**Affiliations:** Latifa Hospital Dubai, UAE; Gruppo Italo Belga, Villa del Rosario and Gemelli Hospitals Università Cattolica, Rome, Italy; Professor Emeritus Department of Obstetrics and Gynaecology, Catholic University Leuven, University Hospital, Gasthuisberg, B-3000 Leuven, Belgium; University of South Australia, Adelaide, Australia; Malzoni Center Naples, Italy; Department of Operative Gynaecology, Federal State Budget Institution V. I. Kulakov Research Centre for Obstetrics, Gynecology, and Perinatology, Ministry of Health of the Russian Federation, Moscow, Russia; and The Department of Reproductive Medicine and Surgery, Moscow State University of Medicine and Dentistry, Moscow, Russia; Endometriosis Centre, Dres. Keckstein Villach, Austria and University Ulm, Ulm, Germany; University of Strasbourg, France

**Keywords:** Deep endometriosis, sensitivity, Bayesian, specificity, test accuracy

## Abstract

The usefulness of a test is determined by the clinical interpretation of its sensitivity and specificity. The pitfalls of a test with a surgical endpoint are described in this article, taking the diagnosis of deep endometriosis by imaging as an example, without discussing the management of deep endometriosis. Laparoscopy is not a 100% accurate “gold standard”. Since it is not performed in women without symptoms, results are valid only for the group of women as specified in the indication for surgery. The confidence limits of accuracy estimations widen when accuracy is lower and when observations are less. Since positive and negative predictive values are inaccurate when prevalence of the disease is low, prevalence figures in the group of women investigated should be available. The accuracy of imaging should be stratified by clinically important aspects such as localisation and size of the lesion. The use of other variables as soft markers during ultrasonographic examination should be specified. It should be clear whether the accuracy of the test reflects symptoms and clinical examination and imaging combined, or whether the accuracy of the added value of imaging which requires Bayesian analysis. When imaging is used as an indication for surgery, circular reasoning should be avoided and the number of symptomatic women not undergoing surgery because of negative imaging should be reported. In conclusion, imaging reports should permit the clinician to judge the validity of the accuracy estimations of a diagnostic test, especially when used as an indication for surgery and when surgery is the gold standard to diagnose a disease.

## Introduction

**Figure 1:**
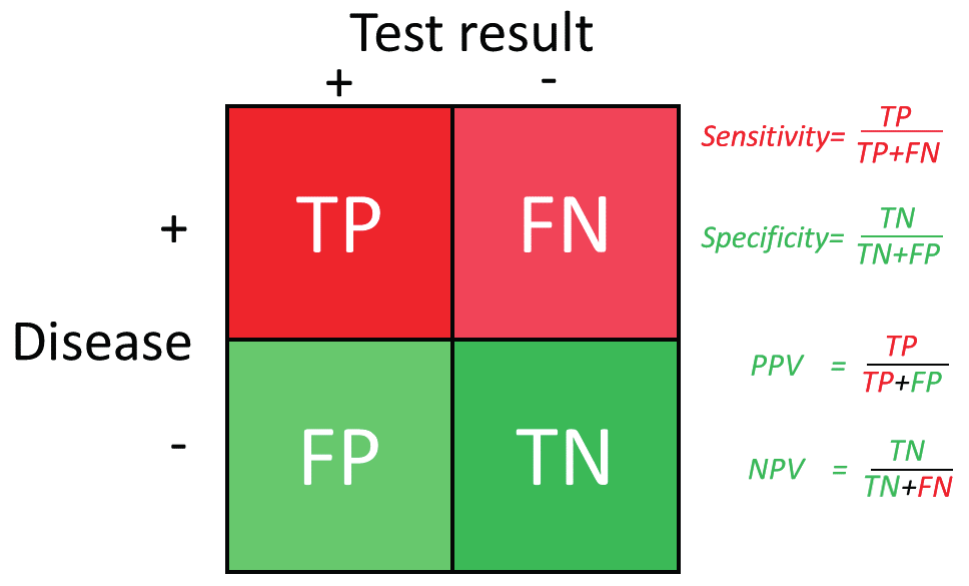
Accuracy of a test. Women with the disease are indicated in red and those without the disease in green. Women with the disease and diagnosed by the test are the true positives (TP); those with a negative test are the false negatives (FN). Women without the disease and a negative test are the true negatives (TN), those with a positive test are the false positives (FP). Sensitivity and specificity thus deal with the population. Sensitivity is the percentage of women with the disease that are diagnosed as having the disease and specificity is the percentage of women who do not have the disease and diagnosed as not having the disease. Positive predictive value (PPV) and negative predictive value (NPV) deal with the test result, and describe the probability that a positive or negative test predicts as to whether a person has or does not have the disease respectively.

The accuracy of a diagnostic test is defined by its sensitivity and specificity (Mallett et al., 2012; Šimundić, 2009). Sensitivity is the percentage of women who have the disease and are diagnosed by the test as having the disease. Specificity is the percentage of women who do not have the disease and are diagnosed as not having the disease (Fig 1). The calculation of sensitivity and specificity thus requires that it is known who has the disease. This needs a 100% accurate “gold standard to make the diagnosis. Clinically, sensitivity and specificity are difficult to translate into decision making. What is clinically important is the probability that somebody with a positive or negative test indeed has or does not have the disease. These are the positive predictive value (PPV) and negative predictive value (NPV) respectively.

The accuracy and the clinical interpretation of a diagnostic test is complicated when used before surgery, and when surgery is needed to confirm the diagnosis. These potential pitfalls of a diagnostic test will be discussed, taking imaging of deep endometriosis as an example. Imaging of endometriosis estimates the presence, localisation, and dimension of deep endometriosis lesions together with information such as adhesions and ureteral involvement. This information is used together with other clinical information such as history, symptoms, clinical exam and eventually other tests in clinical decision making when deciding whether to perform surgery, which surgery will be performed, to plan surgery and to discuss informed consent (Deslandes et al., 2020).

The clinical management of deep endometriosis is much more complex than the diagnostic accuracy of imaging and is outside the scope of this article. Clinical decision making and management are based on a combination of all the information available. In addition, interpretation is guided by experience. Therefore, medicine is considered an art, which is difficult to translate into diagnostic algorithms which can be handled by computers. It is complex and poorly understood how the human brain, rightly or wrongly, combines information and how the relative importance of symptoms, clinical exams and a series of tests are handled together with the age, sex, history, circumstances and other diseases (Banning, 2007). This clinical complexity is apparent when we consider that the clinician needs to take decisions in the individual patient, especially where the randomised clinical trials are known to be poorly suited for rare diseases and multimorbidity (Greenhalgh et al., 2014). Many clinical decisions are therefore often an educated guess based on all available scientific information which does rarely reflect the complexity of the individual patient.

Reports on the accuracy of a diagnostic test, with imaging of deep endometriosis as an example, do not always contain all the information required for a correct clinical interpretation. To help both the authors and the clinicians, we will discuss which information should be provided.

## Absence of a gold standard for the diagnosis of deep endometriosis

Histology, using the definition of endometriosis as ‘endometrium like glands and stroma outside the uterus’(Sampson, 1925) can confirm the presence of endometriosis-like cells, but is not useful as a gold standard for the diagnosis of endometriosis in general and deep endometriosis in particular. Although the discussion whether endometriosis is one or several diseases (Koninckx et al., 2019) is beyond the scope of this article , pathology cannot always differentiate between subtle, typical, cystic and deep endometriosis, and the absence of pathological confirmation does not exclude endometriosis. Especially for subtle lesions, the confirmation by pathology varies with the technique of biopsy taking and of the processing of these tiny samples, as well as the number of slides evaluated. For deep endometriosis, pathology cannot differentiate between the overlapping populations of slightly deeper typical lesions and deep endometriosis (Koninckx et al., 2019). Routine pathology is not suited to evaluate the size, localisation and depth of bowel infiltration, although clinically important for the severity of symptoms and for the difficulty, complication rate and type of surgery which varies from conservative excision to bowel resection (Koninckx et al., 2012).

The laparoscopic diagnosis is considered the gold standard (Machairiotis et al., 2013; Sparić, et al., 2011). However, the diagnostic accuracy varies with the expertise and interests of the surgeon. Also, laparoscopy has difficulty in differentiating between deeper typical lesions, fibrotic plaques and deep endometriosis. In addition, lesions can be missed when located deep under the peritoneal surface, in the upper abdomen or in the sigmoid. Depth of bowel infiltration can only be evaluated accurately by pathology of the bowel resection specimen. Depth of infiltration cannot be judged exactly before surgery or during conservative surgery. The imaging diagnosis of endometriosis before surgery therefore should be evaluated as a test with extreme verification bias since women without symptoms will not undergo a laparoscopy. In addition, the combined accuracy of the symptoms together with the clinical exam and imaging should be estimated with Bayesian inference to evaluate the value of each factor separately (Broemeling, 2011). Similarly, the diagnostic accuracy of a test measuring the depth of bowel wall invasion before surgery should be evaluated as a test with extreme verification bias, since a bowel resection with pathological examination will not be performed in all women. Unfortunately, the required statistical models to estimate the accuracy of tests with several verification biases (such as laparoscopy not being performed in asymptomatic women and not all endometriosis lesions being recognised during laparoscopy) are not yet developed and widely available. Clinically we should realise the potential bias that stricter criteria to limit surgery to women with more severe pain and bigger nodules, resulting in an apparent overestimation of sensitivity and specificity of imaging.

## What is the accuracy of imaging for the diagnosis of deep endometriosis and what is its predictive value?

**Figure 2:**
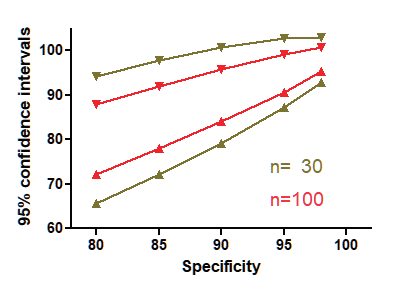
Precision of the sensitivity and specificity estimations increases with the number of observations but decreases when sensitivity or specificity is lower. Confidence intervals are plotted for specificities between 80% and 98% when 30 or 100 samples are investigated respectively.

The sensitivity and specificity of a diagnostic test are independent of the number of observations and of the prevalence of the disease in the population. The precision of the estimations of sensitivity and specificity increases with the number of observations, but decreases when sensitivities and specificities are lower (Figure 2). Therefore it is important to consider confidence intervals of the estimations, e.g. 95% confidence intervals of sensitivity are the sensitivity ±√sensit(1 − sensit)/n ) (Harper and Reeves, 1999). It is important to realise that the 95% confidence limits of a test with 90% sensitivity and 100 observations range from 84% to 96%.

**Figure 3:**
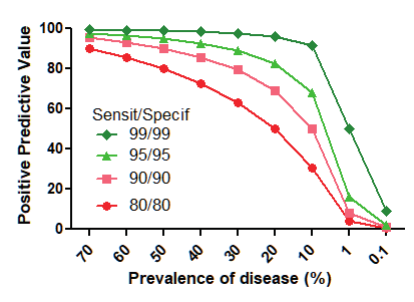
Positive predictive values decrease when the prevalence of the disease decreases and when sensitivity and specificity of the test is less.

Sensitivity and specificity are difficult to translate into clinical decision making. The clinician needs to know when or in what percentage a positive or negative test predicts that somebody does (the PPV) or does not (the NPV) have the disease. However, PPVs and NPVs are strongly influenced by the prevalence of the disease (Fig 3). According to the Bayes’ theorem of conditional probabilities, the PPV=(sensitivity*prevalence)/ (sensitivity*prevalence + (1-specificity)*(1- prevalence)). As an example, a test with 90% sensitivity and 90% specificity will have a 90% PPV if the prevalence of the disease is 50% in the sampled population. Indeed, in 100 people with the disease, 90 true positives will be found, whereas in 100 people without the disease 10 false positive will be found, or a PPV of 90% correctly diagnosed in the 100 positive tests. If the prevalence of the disease is only 1% the PPV drops to 8.2%. In 100 people with the disease 90 are correctly diagnosed, but in the 10,000 without the disease 1000 false positives are found. Even an excellent test with a 99% sensitivity and 99% specificity for a disease with a prevalence of 1% will generate as many true positives as false positives, or a PPV of 50%. This problem is well known but not always taken into account during screening tests, which require a very high level accuracy to avoid too many false positives. Sensitivity and specificity are inter-linked, and sensitivity increases when specificity decreases and vice versa. For numerical endpoints this is obvious from the Receiver Operator Characteristic curves (ROC). Also, for qualitative tests that need judgment and operator experience as for imaging, a more liberal interpretation to diagnose deep endometriosis will increase the number of affected women who are diagnosed. However, this will unavoidably result in a higher number of false positives.

Considering that the accuracy of imaging to diagnose deep endometriosis is rarely evaluated as a test with extreme verification bias (Broemeling, 2011), the reported specificities and sensitivities are valid only for the group of women undergoing a laparoscopy. Since it is unethical to perform laparoscopy in asymptomatic women, the prevalence of disease in the entire population remains unknown. However, the indications for a laparoscopy are variable: pain and other symptoms vary from mild to severe as does the duration of infertility. In addition, the results of imaging contribute to the decision of whether to perform a laparoscopy or not. Diagnostic confidence (Saba et al., 2019) was introduced to address this problem but diagnostic confidence fails to take into account incorrect diagnoses (Ng and Palmer, 2007). Only recently sequential and incremental testing has been evaluated using conditional Bayesian analysis (Chen and Hwang, 2019; Chen, et al., 2019; Hwang and Chen; 2015). Bayesian inference reflects clinical practice if we first consider symptoms, clinical examination and background data (heredity, age, weight etc.) to decide which tests e.g. imaging, will be performed, and if thereafter we decide about the further tests, one of them being a diagnostic laparoscopy. In a previous analysis which considered imaging as an incremental test to diagnose the presence of endometriosis, clinical signs and symptoms were found to be the most important predictive factors (Chen et al., 2019). However, this conclusion should be considered with caution, since the analysis did not take into account different the sizes and localisations of deep endometriosis, and since the data did not permit the evaluation of asymptomatic women. Clinically important today is that ultrasound imaging is generally performed taking into account the clinical examination and symptoms, and that the reported accuracies thus reflect the combined accuracies (Hudelist et al., 2011a; Hudelist et al., 2011b; Hudelist et al., 2009a; Hudelist et al., 2009b). In the absence of a conditional Bayesian analysis, the accuracy of each individual factor cannot be estimated. The importance of deep endometriosis imaging with a silent ureteral obstruction in otherwise asymptomatic women is clinically obvious but requires Bayesian analysis to quantify.

## How to use a diagnostic test of deep endometriosis is a clinical decision

It is a clinical decision how to integrate the results of the imaging of deep endometriosis with all other information (Banning, 2007). In addition, the clinical decision to do surgery varies not only with the estimated probability of the diagnosis, but also with the experience of the surgeon, the clinical strategy, the estimated invasiveness of the disease and risk of complications. Since difficulty of surgery and risk of complications vary with size and localisation (Working group of ESGE/ ESHRE/WES, 2020) the accuracy of imaging need to be stratified by size and localisation. These considerations emphasise the complexity of clinical decision making. We should realise that this complexity is not yet reflected in the mathematical analysis of clinical signs and symptoms and incremental diagnostic tests for diseases with a verification basis (Chen et al., 2019).

Other aspects are important to decide how to use the accuracy of imaging for deep endometriosis. First it should be clear whether, when and how the results of imaging are used in the decision of whether to perform a laparoscopy. The more weight imaging has, the higher the risk of circular reasoning. Second, reported imaging accuracies are generally obtained in tertiary endometriosis referral centres, which have a much higher prevalence of the disease and thus much higher PPVs, than secondary care hospitals. In such referral centres the prevalence of deep endometriosis will often be over 50%, in contrast with a few percent in the population. A test with a sensitivity and specificity of 90% will thus have a PPV of 90% in referral centres, but less than 10% if done in routine practice with a 1% prevalence. The same positive imaging test with 90% sensitivity and specificity for endometriosis thus predicts the disease in referral centres in 9/10 women, but only in 1/10 in non- specialised centres. This bias in the literature risks resulting in inadequate decision making if the PPV of a diagnostic test obtained in referral centres is not updated for the local situation. Third we should realise the huge volume of data which will be required to estimate accuracies of imaging stratified by localisation and size. Stratification will indeed unavoidably decrease the prevalences of each type of lesion and thus lower the PPVs.

Imaging has an important role in the management of deep endometriosis (Alio et al., 2019). The considerations of precision of accuracy estimations, of predictive values and prevalences, of lack of reported accuracies stratified by size and localisation will hopefully help to understand and integrate at the conscious level the complexity of clinical decision making. Imaging is moreover also used for many other purposes than the diagnosis of deep endometriosis. Transvaginal ultrasound does not only take into account the direct visualisation of endometriosis lesions, it also uses indirect signs or ’soft markers’ such as site-specific tenderness and organ mobility (Guerriero et al., 2016; Hudelist et al., 2013), which are subjective and difficult to quantify. In addition, the ultrasonographist is rarely blinded to the clinical symptoms. Unfortunately, it is rarely clear to what extent soft markers contribute to the final diagnosis (Deslandes et al., 2020). In referral centres the feedback between ultrasonographists and surgeons will also refine the use of these soft markers.

## Misuse of statistics for clinical diagnosis and decision making

It must be stressed that the value of a diagnostic test is limited to its specificity and sensitivity, and derived estimations as likelihood ratios. Other statistical outcomes such as significant differences and correlations are important research tools to understand mechanisms. However, this data should not be used for clinical decision making unless validated as a test with a predictive value. As an example, men are significantly taller than women, but height is a poor predictor of sex. That a drug significantly improves patient outcomes does not permit to conclude that this is clinically useful since minimal but clinically insignificant differences become statistically significant when the numbers are high enough. In addition, many medical diagnoses and decisions are multifactorial, and the clinician takes into account the many variables such as antecedents, BMI, other diseases and rare events. However, most of these many variables are individually rarely significant. Similarly, significances of rare events are difficult to calculate because of the prohibitively large numbers required.

## Discussion

Accuracy of preoperative imaging of deep endometriosis must be stratified by size and localization since they predict surgical difficulty and complications, as demonstrated by the ENZIAN classification (Keckstein, 2017; Working group of ESGE/ESHRE/WES, 2020). To interpret clinically the predictive value of imaging, reports should specify the prevalence of lesions stratified by size and localisation in the population investigated. We can anticipate that the higher prevalence in referral centres will result in higher PPV and NPVs than in routine hospitals. The authorsof this article, themselves ultrasonographists estimate a prevalence of around 50% in symptomatic and referred women. Sensitivities and specificities of over 90% thus result in PPVs of over 90% in referral centres.

Reports of accuracy estimations of a test or imaging should permit their clinical interpretation (Table I). Precision of accuracy estimations should be reported since confidence limits are much wider when sensitivity and specificity are lower. To judge the PPV and NPV the prevalence of the disease in the population investigated is required. It should be clear whether the test estimates the accuracy of imaging in women in whom the decision to perform a laparoscopy was already taken clinically, or whether the test estimates the combined accuracy of signs and symptoms and imaging. In future, conditional Bayesian incremental analysis should be done the estimate the value of each parameter. However, this will require much more detailed information on the severity of signs and symptoms and examinations and imaging. The indications to perform a laparoscopy or surgery should be reported in detail since they may vary considerably even between dedicated groups and since the reported accuracies are valid only for this specific indication to perform surgery. The complexity is obvious when considering that as a rule surgery is not performed in asymptomatic women but is mandatory in women with silent ureter obstruction (Muthuppalaniappan et al., 2016). In addition, it should be explained how imaging is used during surgery e.g. to look for deep endometriosis which is not visible by inspection, palpation such as deep lesions around somatic nerves, or multiple bowel lesions. Without exploration, lesions will be missed resulting in false positives. Also, the variable use of soft markers should explicitly be stated, and nodules should be stratified by size and localisation.

**Table I:** Important elements to judge the diagnostic accuracy of imaging of deep endometriosis.

1	The number of women investigated is needed to estimate precision of accuracy estimations.	
2	The prevalence of the disease in the population investigated is needed to estimate PPV.	
3	It should be clear which accuracy of imaging is estimated in women undergoing a laparoscopy	
		Either the combined accuracy of imaging and symptoms and clinical exam
		Or the accuracy of imaging when the decision to do a laparoscopy is taken because of symptoms and clinical exam
		Or the added value of imaging besides symptoms and clinical exam.
		This requires conditional Bayesian inference
4	Since surgery is not performed in all women, the indication for surgery should be clear.	
	a.	If imaging is not used in the indication for surgery, imaging results are valid for the group with a clinical indication for surgery only. Interesting would be to know the number of women with positive imaging not undergoing surgery.
	b.	If imaging is used as an indication for surgery, the test accuracy is valid for this group. It would be nice to know the number of symptomatic women not undergoing surgery because of a negative imaging.
5	It should be specified how imaging is used during surgery, eg to explore invisible lesions	
6	The use of soft markers	
7	Accuracy should be stratified for size and localisation of lesions.	

The diagnostic accuracy of ultrasound imaging and magnetic resonance imaging (MRI) are often compared (Bazot and Darai, 2017). However, this has become a rather academic discussion. Due to the low cost and less invasiveness of ultrasound, which is often used for many other indications in gynaecology, ultrasound imaging has widely become part of the clinical examination. For MRI, the question therefore has become: ‘what is the added value of MRI when the clinical signs and symptoms and the ultrasound evaluation are known?’ Especially for deep endometriosis around larger somatic nerves, or deep in the parametrium, this becomes an important question, but this will require a Bayesian analysis of the added value of MRI.

Although highly likely that preoperative imaging influences surgical intervention, there is a paucity of data on this . This is not surprising since the analysis of the use of imaging in deciding whether to do surgery, which in addition will verify the diagnosis, explains that this is difficult to do. It might even be considered unethical not to use information of imaging when available. In addition, we should realise that using imaging to decide about the type of surgery is circular reasoning, which might become a self-fulfilling prophecy. It is unclear whether more advanced Bayesian inference using conditional probabilities can solve this problem. This again emphasises the complexity of surgical decision making.

## Conclusion

As an overall conclusion, imaging has become a clinically integral part of endometriosis management, notwithstanding the many pitfalls of its diagnostic accuracy. Imaging also helps to judge the extent of disease, adhesions and bowel infiltration which are useful to predict the duration and difficulty of surgery. It is unclear how negative imaging results should be used as an indication for not performing surgery. These problems of the accuracy estimation of imaging of deep endometriosis illustrate the complexity of medical decision making, something which is poorly reflected in the sensitivities and specificities of a test. Moreover, these observations are not limited to imaging of deep endometriosis, but are applicable for all tests with surgery as an endpoint.

## References

[1] Alio L, Angioni S, Arena S (2019). When more is not better: 10 “don’ts” in endometriosis management. An ETIC* position statement. Hum Reprod Open.

[2] Banning M (2008). A review of clinical decision making: models and current research.. J Clin Nurs.

[3] Bazot M, Darai E (2017). Diagnosis of deep endometriosis: clinical examination, ultrasonography, magnetic resonance imaging and other techniques.. Fertil Steril.

[4] Broemeling LD (2011). Bayesian estimation of combined accuracy for tests with verification bias.. Diagnostics.

[5] Chen Z, Hwang BS (2019). A Bayesian semiparametric approach to correlated ROC surfaces with stochastic order constraints.. Biometrics.

[6] Chen Z, Hwang BS, Kim S (2019). A correlated Bayesian rank likelihood approach to multiple ROC curves for endometriosis.. Stat Med.

[7] Deslandes A, Parange N, Childs JT (2020). Current status of transvaginal ultrasound accuracy in the diagnosis of deep infiltrating endometriosis before surgery: A systematic review of the literature.. J Ultrasound Med.

[8] Greenhalgh J, Howick J, Maskrey N (2014). BMJ. Evidence based medicine: a movement in crisis.

[9] Guerriero S, Condous G, Van Den Bosch T (2016). Systematic approach to sonographic evaluation of the pelvis in women with suspected endometriosis including terms, definitions and measurements: a consensus opinion from the International Deep Endometriosis Analysis (IDEA) group.. Ultrasound Obstet Gynecol.

[10] Harper R, Reeves B (1999). Reporting of precision of estimates for diagnostic accuracy: a review.. BMJ.

[11] Hudelist G, Ballard K, English J (2011). Transvaginal sonography vs. clinical examination in the preoperative diagnosis of deep infiltrating endometriosis. Ultrasound Obstet Gynecol.

[12] Hudelist G, English J, Thomas AE (2011). Diagnostic accuracy of transvaginal ultrasound for non-invasive diagnosis of bowel endometriosis: systematic review and meta-analysis.. Ultrasound Obstet Gynecol.

[13] Hudelist G, Fritzer N, Staettner S (2013). Uterine sliding sign: a simple sonographic predictor for presence of deep infiltrating endometriosis of the rectum.. Ultrasound Obstet Gynecol.

[14] Hudelist G, Oberwinkler KH, Singer CF (2009). Combination of transvaginal sonography and clinical examination for preoperative diagnosis of pelvic endometriosis.. Hum Reprod.

[15] Hudelist G, Tuttlies F, Rauter G (2009). Hum Reprod. Can transvaginal sonography predict infiltration depth in patients with deep infiltrating endometriosis of the rectum.

[16] Hwang BS, Chen Z (2015). An integrated Bayesian nonparametric approach for stochastic and variability orders in ROC curve estimation: An application to endometriosis diagnosis.. J Am Stat Assoc.

[17] Keckstein J The ENZIAN classification of Deep Infiltrating Endometriosis In Mettler L, Keckstein J and Meinhold-Heerlein I (eds) Endometriosis: A concise practical guide to current diagnosis and treatment Endopress, Tüttlingen 2017: 44-56

[18] Koninckx PR, Ussia A, Adamyan L (2012). Deep endometriosis: definition, diagnosis, and treatment.. Fertil Steril.

[19] Koninckx PR, Ussia A, Adamyan L (2019). Pathogenesis of endometriosis: the genetic/epigenetic theory.. Fertil Steril.

[20] Machairiotis N, Stylianaki A, Dryllis G (2013). Extrapelvic endometriosis: a rare entity or an under diagnosed condition?. Diagnostic Pathology.

[21] Mallett S, Halligan S, Thompson M (2012). Interpreting diagnostic accuracy studies for patient care.. BMJ.

[22] Muthuppalaniappan VM, Wiles KS, Mukerjee D (2016). Silent obstruction in a young woman with systemic lupus erythematosus: a case report and literature review on kidney injury from ureteral endometriosis.. Postgrad Med.

[23] Ng CS, Palmer CR (2007). Analysis of diagnostic confidence and diagnostic accuracy: a unified framework.. Br J Radiol.

[24] Saba L, Ajossa S, Ledda G (2019). Br J Radiol. Does the clinical information play a role in the magnetic resonance diagnostic confidence analysis of ovarian and deep endometriosis.

[25] Sampson JA (1925). Heterotopic or misplaced endometrial tissue. Am J Obstet Gynecol.

[26] Šimundić AM (2009). Measures of diagnostic accuracy: Basic definitions.. EJIFCC.

[27] Sparić R, Hudelist G, Keckstein J (2011). Diagnosis and treatment of deep infiltrating endometriosis with bowel involvement: a case report.. Srp Arh Celok Lek.

[28] Keckstein J, Becker CM (2020). Recommendations for the surgical treatment of endometriosis. Part 2: deep endometriosis. Facts Views Vis Obgyn.

